# Accurate UAV Small Object Detection Based on HRFPN and EfficentVMamba

**DOI:** 10.3390/s24154966

**Published:** 2024-07-31

**Authors:** Shixiao Wu, Xingyuan Lu, Chengcheng Guo, Hong Guo

**Affiliations:** 1 School of Information Engineering, Wuhan Business University, Wuhan 430056, China; wushixiao@whu.edu.cn; 2 Key Laboratory of Computer Vision and System, Ministry of Education, Tianjin University of Technology, Tianjin 300384, China; 3 School of Information Engineering, Wuhan College, Wuhan 430212, China; 4 School of Electronic Information, Wuhan University, Wuhan 430072, China; 5 School of Computer Science and Engineering, Tianjin University of Technology, Tianjin 300384, China; 20150725230@mail.sdufe.edu.cn

**Keywords:** small object detection, deep learning, HRNet, Mamba, YOLO, feature fusion

## Abstract

(1) Background: Small objects in Unmanned Aerial Vehicle (UAV) images are often scattered throughout various regions of the image, such as the corners, and may be blocked by larger objects, as well as susceptible to image noise. Moreover, due to their small size, these objects occupy a limited area in the image, resulting in a scarcity of effective features for detection. (2) Methods: To address the detection of small objects in UAV imagery, we introduce a novel algorithm called High-Resolution Feature Pyramid Network Mamba-Based YOLO (HRMamba-YOLO). This algorithm leverages the strengths of a High-Resolution Network (HRNet), EfficientVMamba, and YOLOv8, integrating a Double Spatial Pyramid Pooling (Double SPP) module, an Efficient Mamba Module (EMM), and a Fusion Mamba Module (FMM) to enhance feature extraction and capture contextual information. Additionally, a new Multi-Scale Feature Fusion Network, High-Resolution Feature Pyramid Network (HRFPN), and FMM improved feature interactions and enhanced the performance of small object detection. (3) Results: For the VisDroneDET dataset, the proposed algorithm achieved a 4.4% higher Mean Average Precision (mAP) compared to YOLOv8-m. The experimental results showed that HRMamba achieved a mAP of 37.1%, surpassing YOLOv8-m by 3.8% (Dota1.5 dataset). For the UCAS_AOD dataset and the DIOR dataset, our model had a mAP 1.5% and 0.3% higher than the YOLOv8-m model, respectively. To be fair, all the models were trained without a pre-trained model. (4) Conclusions: This study not only highlights the exceptional performance and efficiency of HRMamba-YOLO in small object detection tasks but also provides innovative solutions and valuable insights for future research.

## 1. Introduction

Under the rapid development of deep learning, significant progress has been made in object detection technology. Some general object detection algorithms perform excellently on mainstream large-scale datasets [1,2,3]. However, these algorithms often fall short when it comes to small object datasets like UAVs. UAVs operate in a dynamic and diverse low-altitude setting, characterized by numerous obstacles and impediments (like trees, buildings, etc.) as well as unpredictable factors such as weather, lighting, noise, kites, and birds; small objects for UAVs (typically defined as targets with pixels fewer than 32 × 32) are frequently disregarded, thereby intensifying the challenge of detection [4].

Small object datasets have the following characteristics: (1) difficulty in feature extraction, as small objects occupy a small area of the image, resulting in fewer effective features available for detection; (2) high requirements for localization accuracy, where small objects may be distributed across various regions of the image, including corners, and may be occluded by larger objects, while also being susceptible to image noise; (3) an uneven proportion and distribution of small objects, such that they may be densely packed or scattered throughout the image; and (4) imbalanced samples, which refers to a situation where the number of samples of different categories in a dataset varies greatly. Sample imbalance in the context of UAV data can pose significant challenges, especially in applications such as object detection, anomaly detection, and land cover classification. UAVs often collect vast amounts of data, but certain classes (e.g., specific objects or types of terrain) may be underrepresented. Addressing sample imbalance is crucial for building accurate and reliable models in these applications. Leveraging a multi-scale feature fusion approach can effectively address the issue of increasing small objects by detecting objects of various sizes through distinct feature levels. This study aims to address the issue of detecting small objects in UAV imagery through improved feature extraction.

**Figure 1:**
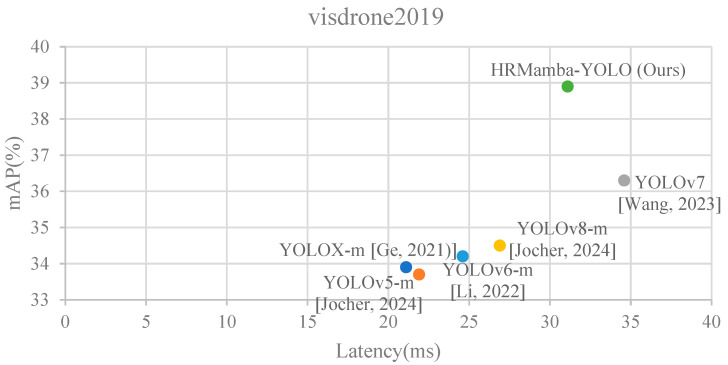
Comparison of various methods [2,5,6,7].

Inspired by HRNet, Mamba, and YOLO architecture, this research proposes HRMamba-YOLO, a small object detection algorithm. It integrates Mamba’s long-range modeling capabilities, HRNet’s high-resolution representation learning, and YOLO’s fast and accurate detection capabilities, aiming to efficiently detect small objects (partial results can be seen in Figure 1). 

The main contributions of this research can be summarized as the following:

(1) We introduce a module named Double SPP designed to enhance a feature extraction network’s performance and adapt more effectively to small object detection tasks. This module, by incorporating an additional spatial pyramid pooling layer, improves the network’s ability to extract features across different scales.
(2) In this study, we also developed EMM and FMM, both based on efficient 2D scanning (ES2D) design. They enhance feature representation and a network’s ability to capture contextual information by processing multi-scale feature information.
(3) Additionally, we redesigned a Multi-Scale Feature Fusion Network, termed the HRFPN. Based on HRNet architecture, this network improves feature information at all scales through intra-scale feature processing and cross-scale feature fusion.
(4) Finally, building on the proposed modules and networks, we established a novel small object detection network, HRMamba-YOLO. This network combines the strengths of Mamba, HRNet, and YOLO to achieve the high-performance detection of small objects. From Figure 1, it can be observed that although the speed advantage decreased, the mAP of the proposed algorithm on the VisDrone dataset reached the highest mAP in a comparative range.

## 2. Related Work

### 2.1. Small Object Detection

Guangyi Tang Tang conducted a survey on object detection for UAVs using deep learning techniques [8]. He offered a review of UAV evolution and consolidated deep learning approaches in UAV object detection. Furthermore, he examined critical challenges in UAV object detection, including but not limited to small object detection, identifying objects against intricate backgrounds, handling object rotation and scale variations, and tackling categorical imbalance issues. The method of multi-scale feature fusion can effectively address the challenge of rising small objects by detecting objects of varying sizes using diverse levels of features. In early UAV object detection research, Sevo and Avramovic demonstrated the effective integration of CNNs into aerial image object detection algorithms [9]. Sommer et al. utilized a Fast R-CNN and Faster R-CNN for vehicle detection in aerial images [10]. Unfortunately, the algorithms proposed in these two papers could not achieve real-time performance. Despite the generalization ability of CNNs, extracting effective features from small objects that occupy limited pixels in images poses a challenge. Additionally, CNNs may not be robust enough to handle rotation, occlusion, illumination, and other variations in small objects, leading to potential false positives or false negatives. To enhance small object detection performance, Liu et al. introduced UAV-YOLO, which optimized the ResBlock in darknet by connecting two ResNet units with equal width and height [11]. The fps of UAV-YOLO is only 20, which is a bit slow. Zeng et al. incorporated a hybrid attention mechanism involving coordinate-related attention and multi-layer feature fusion to effectively differentiate foreground and background features in aerial images, enriching the semantic information of shallow features [12]. This algorithm is based on YOLOv5 and demonstrated improved accuracy and detection speed on the VisDrone2020 dataset, though it may not be fit for other datasets. Qian et al. combined Haar-like features with the MobileNet-SSD algorithm, employing a top–down and horizontal connection approach to building a feature pyramid with high resolution and strong semantics, facilitating multi-scale UAV feature representation and detection [13]. The algorithm improved recognition accuracy but did not address the discussion of an mAP metric. Tian et al. introduced the DNOD method, which utilizes a VGG network for feature map extraction from UAV images and the integrated location information of suspected regions for secondary identification, reducing false negatives in small object detection [14]. They further validated the algorithm’s reliability and effectiveness by combining Yolov4 and EfficientDet-D7, but they did not mention other algorithms. Additionally, there was no description of the detection speed on the Visdrone dataset.

The algorithms mentioned above utilize one-stage object detection algorithms to tackle the issue of small object detection. However, many studies have employed two-stage object detection algorithms to address small object detection challenges. Jinshan Cao et al. proposed a GhostConv-based lightweight YOLO network specifically tailored for the detection of small objects in UAV images [15]. This method was based on YOLOv5 and used the VisDrone-DET2021 dataset, though it may not be well suited for the YOLOv8 model and other datasets. To enhance the detection performance of small objects in UAV images, Zhu et al. introduced transformer prediction heads (TPHs) and a convolutional block attention model into the YOLOv5 network, creating a TPH-YOLOv5 network [16]. TPH-YOLOv5 was based on the design of YOLOv5 and had a significantly improved mAP, but there was no discussion on its speed. Sun et al. proposed a real-time small object detection (RSOD) method for UAV-based traffic monitoring based on YOLOv3 [17]. The RSOD’s mAP improved significantly, but the speed was only 28. Li et al. explored an image cropping strategy and presented a density map-guided object detection network (DMNet) that leveraged spatial and contextual information between objects to enhance detection accuracy [18]. The DMNet’s mAP was a little lower, at only 28.2. Liu et al. developed the MYOLO-lite network, a lightweight variant of YOLOv4 with a MobileNet as the backbone, effectively reducing network parameters and computational complexity to meet the speed requirements in UAV object detection applications [19]. This paper reported results for only one class and did not been validate the network on a public dataset. Zhang et al. introduced SlimYOLOv3, a pruned version of YOLOv3, achieving twice the detection speed while maintaining the detection accuracy [20]. The mAP of SlimYOLOv3-SPP3-95 was only 21.2.

### 2.2. Feature Pyramid Network

Introducing a pyramid network enables an object detection algorithm to better handle multi-scale targets, enhance semantic information, and improve detection performance, making the algorithm perform better in object detection tasks. Common feature pyramid networks are described as follows.

A Feature Pyramid Network (FPN) is a deep neural network architecture widely used in object detection tasks [21]. It aims to address the challenge of detecting objects at different scales by constructing a feature pyramid from a single-scale input. An FPN enhances performance by incorporating multi-scale features at different levels of the network, enabling the detection of objects of various sizes.

A Bidirectional Feature Pyramid Network (BiFPN) is an extension of the FPN architecture but introduces bidirectional connections between different levels of the feature pyramid [22]. By incorporating both top–down and bottom–up pathways, a BiFPN enhances the flow of information across different scales, leading to improved object detection performance. In EfficientDet, the BiFPN emphasizes bidirectional cross-scale connections and weighted feature fusion. In the BiFPN, connections between feature layers are bidirectional, and the introduction of weights differentiates the importance of different feature layers for more effective feature fusion.

A Path Aggregation Network (PANet) is a network architecture designed for, for instance, segmentation tasks, which involve both object detection and pixel-wise segmentation. A PANet introduces a path aggregation module to aggregate features from different levels of the feature pyramid, improving the quality of feature representations and enhancing the segmentation accuracy [23].

A Recursive Feature Pyramid Network (Recursive-FPN) is a variant of the FPN architecture that incorporates recursive connections between different levels of the feature pyramid [24,25]. By recursively aggregating features from multiple scales, a Recursive-FPN aims to capture rich contextual information and enhance the detection of objects across various scales.

However, while these classic multi-scale feature fusion methods perform well in general object detection, they may not be fully applicable to small object detection scenarios [26,27]. Therefore, this study proposes a new feature fusion method, HRFPN, designed to address the specific challenges of small object detection and effectively learn more contextual information. The HRFPN combines the HRNet and high-resolution representation learning with advanced feature fusion concepts, providing a new solution for small object detection.

### 2.3. State Space Model on Visual Recognition

In the field of deep learning, State Space Models (SSMs) have emerged as a key technology for handling long-range dependencies in sequential data, demonstrating significant application potential [28,29,30]. Inspired by continuous control systems and combined with HiPPO initialization, the LSSL model has showcased the immense potential of SSMs, despite challenges in computational complexity and storage requirements. With the introduction of the Structured State Spaces for Sequence Modeling (S4) model, significant performance improvements have been achieved through optimized parametric structures and the adoption of normalization methods. Subsequently, various innovative SSM architectures, such as complex diagonal structures and selection mechanisms, have provided notable advantages across multiple application scenarios.

In the realm of visual recognition, Vision Mamba (Vim) has pioneered the introduction of a Bidirectional State Space Model to enhance visual representation learning. Compared to DeiT, Vim has achieved superior performance across multiple visual tasks while maintaining high efficiency [31]. VMamba addresses global perception in a two-dimensional image space through the introduction of a Cross-Scan Module (CSM), maintaining linear computational complexity and significantly enhancing input scaling efficiency. Models like MedMamba, Swin-UMamba, and U-Mamba are specifically tailored for medical image tasks, combining the local feature extraction capabilities of convolutional layers with the long-range dependency-capturing abilities of SSMs, effectively modeling different modalities of medical images through the SS-Conv-SSM module. EfficientVMamba, through efficient skip sampling and regrouped, dilated, selective scanning methods, integrates SSM blocks with convolutional branches, significantly reducing computational complexity and achieving competitive results across multiple visual tasks [32,33,34,35]. 

Against this backdrop, our proposed HRMamba-YOLO model ingeniously integrates SSMs with lightweight object detection algorithms, specifically optimized for small object detection, resulting in a marked performance enhancement.

## 3. Methods

### 3.1. Overall Architecture

**Figure 2:**
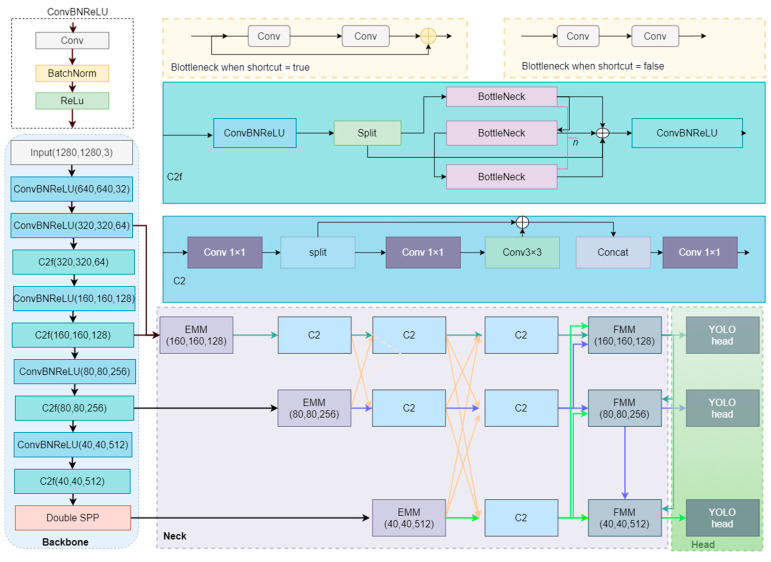
The HRMamba-YOLO architecture (In neck, the orange arrows represent the fusion of different feature maps, and the other color arrows represent the forward transfer of feature maps with different resolutions).

The detailed structure of the HRMamba-YOLO architecture proposed in this article is shown in Figure 2. The network is primarily composed of five parts: a backbone, an EMM, an HRFPN, an FMM, and a YOLO Head.

**Backbone**: We employed the YOLOv8 backbone as the fundamental feature extraction network [5]. Following an initial stem downsampling operation, the backbone was divided into four main stages, outputting feature maps at 1/4, 1/8, 1/16, and 1/32 scales, providing inputs for the subsequent feature fusion network. Additionally, we introduced a Double SPP module at the end of the backbone to enhance the receptive field and enrich the features. **In the backbone, there are four C2f modules from top to bottom, where the values of n are 1, 2, 2, and 1, respectively**.

**EMM**: The primary purpose of an EMM is to expand a mode’s global receptive field before feature fusion, enhancing the capture of extensive contextual information. The EMM mainly targets feature maps at 1/8, 1/16, and 1/32 scales and combines the features from corresponding scales with those of larger scales.

**HRFPN**: An HRFPN is a feature fusion network designed based on HRNet architecture, specifically optimized for small object detection tasks. The characteristic of HRNet is maintaining high-resolution feature maps throughout a network, thus improving the detection efficiency of small objects. The HRFPN is responsible for implementing efficient feature fusion across 1/8, 1/16, and 1/32 scales. In this article, HRFPN refers to the part of the neck excluding the EMM module.

**FMM**: An FMM also applies Mamba technology, effectively leveraging useful information from various scales to output feature maps of the current scale.

**YOLO Head**: We followed the detection head design of YOLOv8, performing classification and position prediction analyses on the processed feature maps across three scales to accurately identify targets within the image.

### 3.2. Double SPP in Backbone

**Figure 3:**
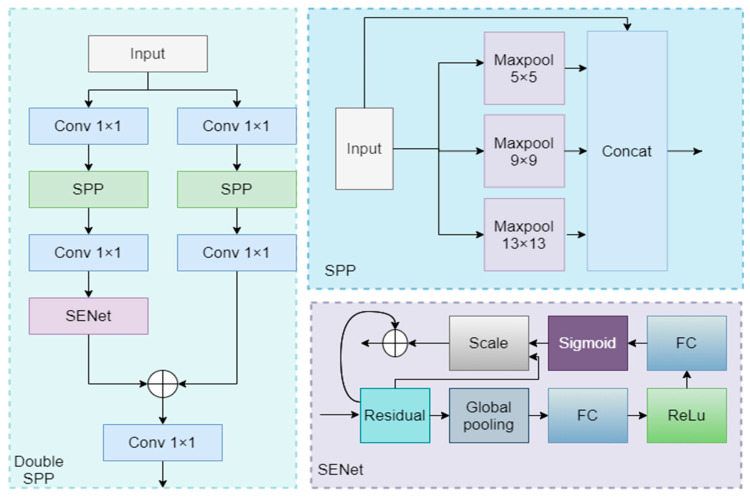
Double SPP.

The Double SPP module (Figure 3), designed to enhance the accuracy of small object detection, extends the traditional SPP technique. By performing pooling operations at different scales, Double SPP captures multi-scale contextual information, which is crucial for the effective detection of small objects. Double SPP employs two independent SPP modules operating on two separate branches, enhancing the extraction of multi-scale features and facilitating a more detailed capture of small object characteristics [36].

One branch integrates the Squeeze-and-Excitation (SE) attention module, which dynamically adjusts channel weights by evaluating the importance of feature channels, achieving feature recalibration [37]. In small object detection, the SE module is particularly critical as it guides the network to focus on features related to small objects and suppresses irrelevant information, thereby improving detection accuracy. Deploying the SE module on one branch allows the network to more effectively extract subtle features of small objects, enhancing performance in subsequent detection stages.

The feature maps from both branches are merged through an addition operation, a strategy that enables the network to synthesize the strengths of both branches, producing richer and more robust feature representations. For small object detection, this approach provides the model with more comprehensive contextual information, helping to reduce the probability of false positives and misses.

### 3.3. Neck

#### 3.3.1. EfficientVMamba

SSMs are continuous system models inspired by the classical Kalman filter model. These models map one-dimensional functions or sequences to hidden states through an evolution parameter and a projection parameter. The dynamics of the system are described by the following set of equations:

(1) $$h^{\prime }(t)=Ah(t)+\mathrm{Bw}(t)$$

(2) y(t)=Ch(t)

where $\bar{A}\in {ℜ}^{N\times N}$, $\bar{B}\in {ℜ}^{D\times N}$, and $\bar{C}\in {ℜ}^{D\times N}$. 

Discretization involves converting continuous differential equations into discrete functions to match the sampling frequency of the input signal, thereby enhancing computational efficiency. Continuous parameters *(A, B)* can be discretized using the zero-order hold method at a specified sampling interval $\Delta \in {ℜ}^{D}$:

$$\bar{A}=e^{\Delta A},$$

$$\bar{B}=(e^{\Delta A}-I)A^{-1}B,$$

(3) $$\bar{C}=C,$$

$$\bar{B}\approx (\Delta A){\left(\Delta A\right)}^{-1}AB=\Delta B,$$

$$h(t)=\bar{A}h(t-1)+\bar{B}x(t),$$

$$y(t)=\bar{C}h(t).$$

**Figure 4:**
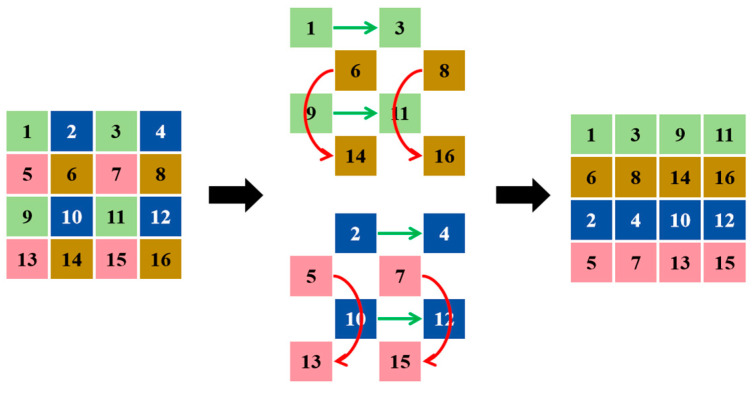
Description of ES2D (ES2D adopts a strategy of scanning forward vertically and horizontally while skipping patches and maintaining the number of patches unchanged. Their efficient visual state space (EVSS) block comprises a convolutional branch for local features, uses ES2D as the SSM branch for global features, and all branches end through a squeeze–excitation block. They employ EVSS blocks for the horizontal direction (marked with green lines), while opting for inverted residual blocks for the vertical direction (marked with red lines), to enhance the capture of global representations).

**Figure 5:**
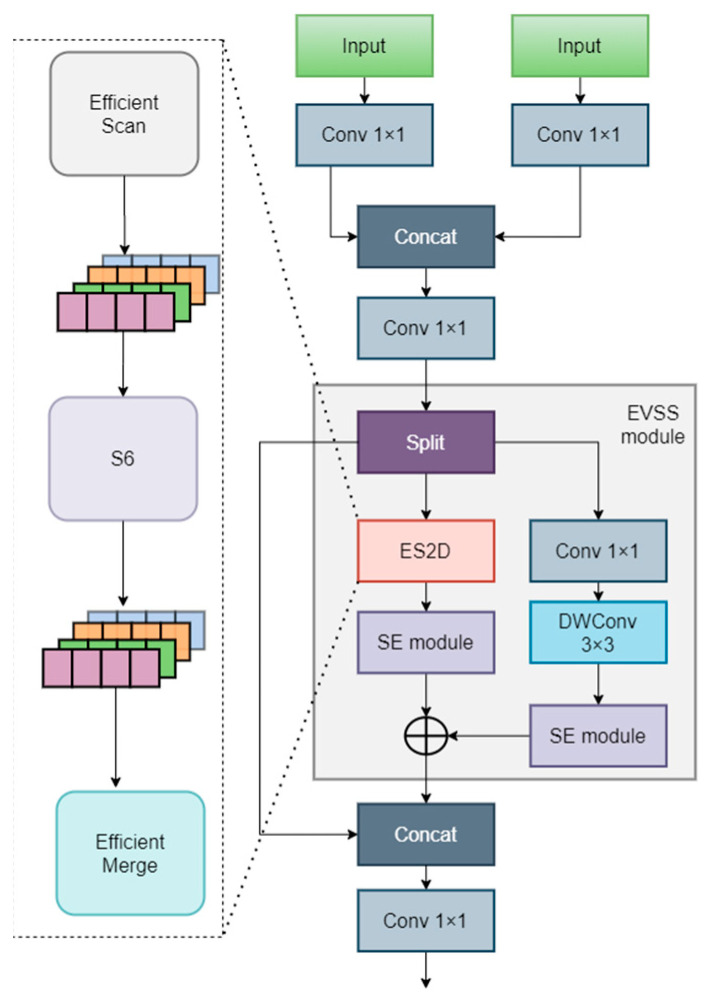
The EMM module.

ES2D is an advanced two-dimensional scanning method designed to address the computational bottlenecks in visual tasks (Figure 4). The process is illustrated in Figure 5. Traditional SSMs face limitations when dealing with large-scale visual tasks due to their global information extraction, which has a time complexity of *O(N^2^)*. ES2D innovatively alleviates this issue by introducing a selective scanning strategy based on dilated convolutions and an efficient skip sampling mechanism, effectively reducing the number of tokens to be processed in the spatial dimension.

ES2D organizes data blocks by omitting the sampling step and then performing intra-group traversal. In this method, the input feature map ($X\in R^{C\times H\times W}$) is divided into multiple patches, which are then selectively scanned through skip sampling with a stride (p). The ES2D does not scan entire blocks crosswise; instead, it skips scanning blocks with a stride of p, segmenting them into selected spatial dimension features ${\left\{O_{i}\right\}}_{i=1}^{4}$. This process can be expressed as the following:

$$O_{i}↔X[:,m::p,n::p],$$

(4) $${\left\{{\tilde{O}}_{i}\right\}}_{i=1}^{4}\leftarrow SS2D({\left\{O_{i}\right\}}_{i=1}^{4})$$

where $O_{i},{\tilde{O}}_{i}\in {ℜ}^{C\times \frac{H}{P}\times \frac{W}{P}}$, and *((m,n))* identifies the sampling starting point, ensuring interval sampling across the feature map. The operation [:, m::p, n::p] represents the slicing of the matrix for each channel, starting at m in height *(H)* and n in width *(W)*, and skipping p steps. The skip sampling of the local receptive field reduces computational complexity by selectively scanning smaller blocks of the feature map. With a stride of p, the authors sample blocks of size *(C, H/p, W/p)* at intervals of p. Compared to *(C, H, W)* in SS2D, the amount of data processed in each scan and merge operation is reduced from *N* to *N/P*^2^, improving the efficiency of feature extraction. These patches, after being processed by SS2D, form a new feature set ${\left\{{\tilde{O}}_{i}\right\}}_{i=1}^{4}$, which is ultimately merged back into the output feature map *(Y)* as the following:

$$Y[:,m::p,n::p]\leftarrow merge({\tilde{O}}_{i}),$$

(5) $$\left(m,n)=(\left\lfloor \frac{1}{2}+\frac{1}{2}\sin(\frac{\pi }{2}(i-2)\right\rfloor ,\left\lfloor \frac{1}{2}+\frac{1}{2}\cos(\frac{\pi }{2}(i-2)\right\rfloor \right)$$

By employing this strategy, ES2D reduces the complexity from *O(N^2^)* to *O(N/p^2^)*, maintaining a global receptive field while significantly decreasing computational demands. This increase in computational efficiency is crucial for handling large datasets, and by merging the processed feature patches, ES2D can reconstruct the global structure of the feature map, capturing more comprehensive contextual information.

Mamba improves the performance of the SSM by introducing a Selective State Space (S6), which allows continuous parameters to vary with input, enhancing selective information processing between sequences. This extends the discretization process through a selection mechanism.

(6) $$\bar{B}=S_{B}(x),$$

(7) $$\bar{C}=S_{c}(x),$$

(8) $$\Delta =\tau_{A}(Parameter+S_{A}(x)).$$

Here, $S_{B}(x)$ and $S_{c}(x)$ project the input *x* into an N-dimensional space, where $S_{A}(x)$ expands a D-dimensional linear projection to the necessary dimensionality.

#### 3.3.2. EMM

This study introduces an efficient neural network module named EMM, whose core design philosophy is to enhance detection performance by integrating multi-scale feature fusion techniques with attention mechanisms. The specific structure is shown in Figure 5.

Specifically, EMM takes high-resolution feature maps and corresponding scale feature maps as inputs. It adjusts the high-resolution input to match the current scale through downsampling operations. Subsequently, both inputs undergo 1 × 1 convolutions to transform the features, which are then merged through a concatenation (Concat) operation. The merged features are further processed by another 1 × 1 convolution for dimensionality reduction. For an efficient visual state space (EVSS) block, the features are split in the channel dimension into two parts: one part undergoes a residual connection, while the other is fed into a dual-branch structure. In the dual-branch structure, the first branch reshapes the global feature map using the ES2D module to capture contextual information at a lower cost, followed by an SE attention module for feature recalibration. The second branch implements feature transformation and recalibration through a 1 × 1 convolution, a depthwise separable convolution (DWConv 3 × 3), and an SE attention module. Finally, the outputs of both branches are combined and concatenated with the previous residual connection, and a final 1 × 1 convolution produces the ultimate feature map.

In ES2D, reorganizing the global spatial feature map involves combining processed blocks to reconstruct the global structure of the feature map. This integration captures a broader context, balancing local details and the global context in feature extraction. The EVSS block aims to synergistically fuse global and local feature representations while maintaining computational efficiency. It leverages an ES2D modified by squeezing to capture global information and customizes a convolutional branch to extract crucial local features, with both branches going through subsequent SE blocks.

#### 3.3.3. High-Resolution Feature Pyramid Network

**Figure 6:**
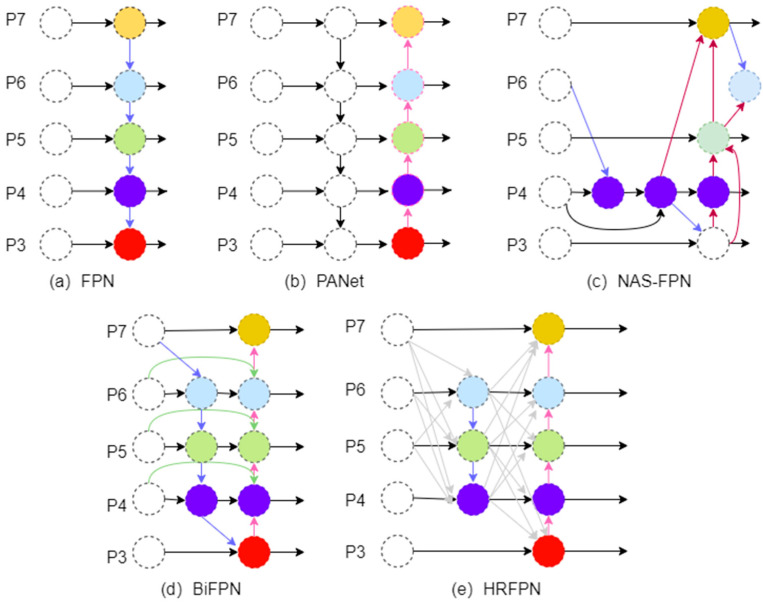
Comparison of various feature pyramid networks.

The core of our method lies in designing and implementing a novel feature fusion network, dubbed the HRFPN, inspired by the state-of-the-art HRNet architecture. The specific structure is shown in Figure 6. Our goal is to enhance small object detection by effectively integrating features of different scales, leveraging unique attributes inherent to each scale, such as texture detail and receptive field size.

**Cross-Scale Feature Fusion**: HRFPN seamlessly integrates features at 1/8, 1/16, and 1/32 scales through its core component, the cross-scale feature fusion module. The module’s fusion strategy maximizes the complementarity of features at different resolutions, combining fine-scale textural details with coarse-scale comprehensive representations to improve small object recognition.

**Intra-Scale Feature Processing**: We deployed a C2Layer and 1 × 1 convolution layers within each scale to refine feature information, ensuring the network captures small object details. This step crucially enhances the network’s sensitivity and recognition capabilities for small-scale objects.

**Figure 7:**
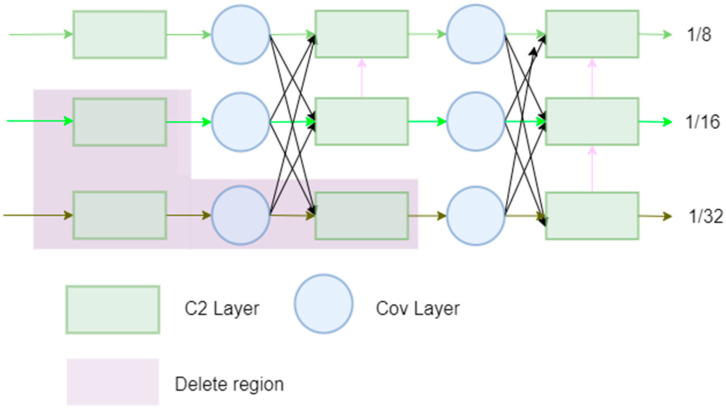
The specific structure of the HRFPN.

**Implementation Details:** For object detection at 1280 × 1280 resolution, we particularly focused on processing larger scale features. Thus, we increased the convolutional processing of larger-scale features to ensure the network’s capacity to distinguish small objects. Concurrently, we adopted HRNetv2’s strategies for network simplification to enhance efficiency, as indicated by the purple areas in Figure 7, which mark the parts we pruned.

Figure 6 provides a comparison of various feature pyramids, with the pyramid network proposed by us being HRFPN. FPN layers transmit strong semantic features from top to bottom, while the feature pyramid conveys strong localization features from bottom to top. These two approaches work together, aggregating parameters from different backbone layers to various detection layers. PANet is an improvement over FPN, designed to add a bottom–up pyramid after FPN. PANet introduces path aggregation, which combines shallow feature maps (low-resolution but weak semantic information) and deep feature maps (high-resolution but rich semantic information), and it passes feature information along specific paths, conveying strong localization features from lower layers upward. NAS-FPN utilizes a reinforcement learning-based search algorithm to discover optimal network structures for feature fusion. This method automatically finds better FPN architectures, optimizing both accuracy and efficiency. The BiFPN uses bidirectional cross-scale connections with learnable weights to efficiently combine features from different resolutions. For the COCO validation set, the AP values for repeated top–down FPN, repeated FPN + PANet, NAS-FPN, and BiFPN are 42.29, 44.08, 43.16, and 44.39, respectively. HRNet-W48 can achieve an AP of 76.3, so we chose HRNet as the direction for improvement.

In Figure 7, the pink arrows represent the new connections added in the HRFPN based on HRNetv2.

#### 3.3.4. Fusion Mamba Module

**Figure 8:**
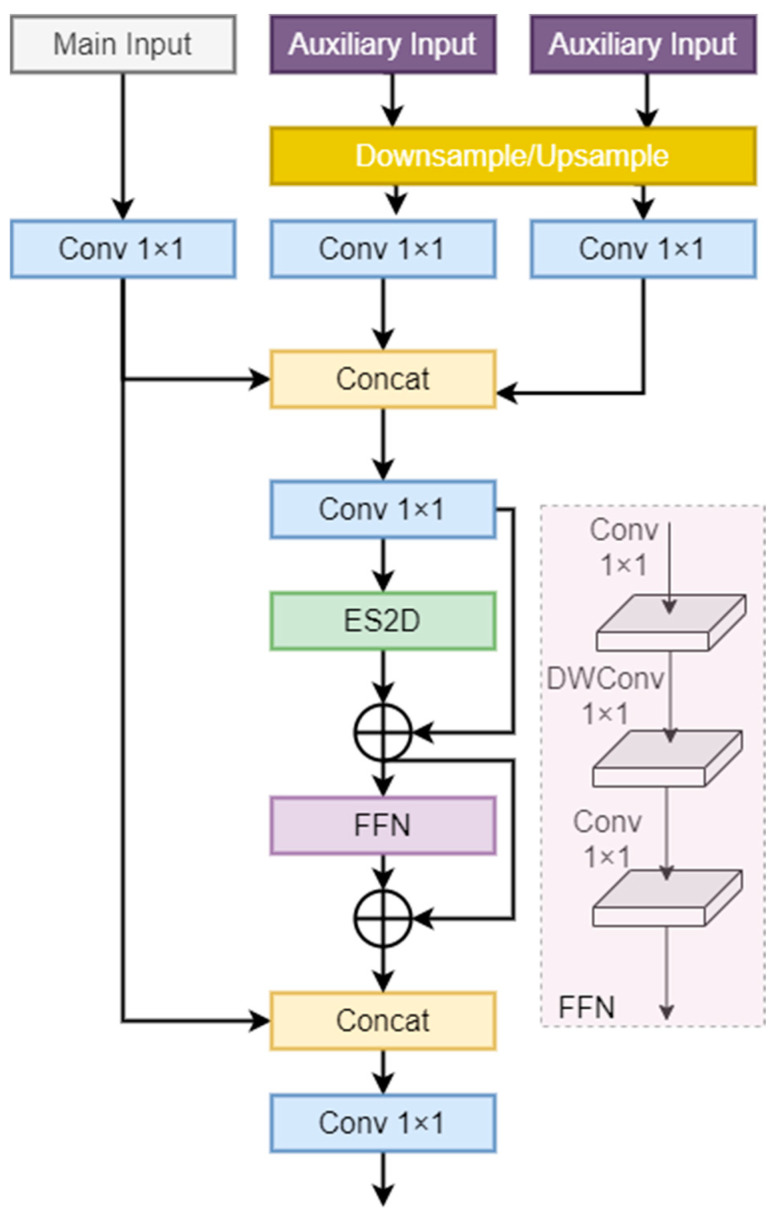
The FMM module.

The FMM is positioned between the HRFPN and the YOLO Head, responsible for final feature fusion and processing. Figure 8 showcases the detailed structure of the FMM. The FMM receives feature information from the current scale, supplemented by features from two other scales. First, the auxiliary inputs are adjusted to the same resolution as the main input through upsampling or downsampling, followed by a transformation using 1 × 1 convolution. These three transformed inputs are concatenated in the channel dimension, followed by another transformation using 1 × 1 convolution. Subsequently, the sequence is processed through ES2D and a feedforward network (FFN), with residual connections introduced to prevent information loss. The processing result is concatenated with the initial main input, followed by a final transformation and output.

FMM is designed to optimize feature representation, enhancing small object detection performance by considering the relevance of current features with cross-scale features. The processing of current features involves multiple applications of 1 × 1 convolutions for linear transformations and a residual connection with the initial input before the final output to ensure information integrity. Cross-scale feature processing integrates information from other scales, efficiently enhancing the interaction between the main and related features through ES2D and FFN. This approach deeply explores the relationships between different features and captures long-distance dependencies across multiple scale resolutions.

**Figure 9:**
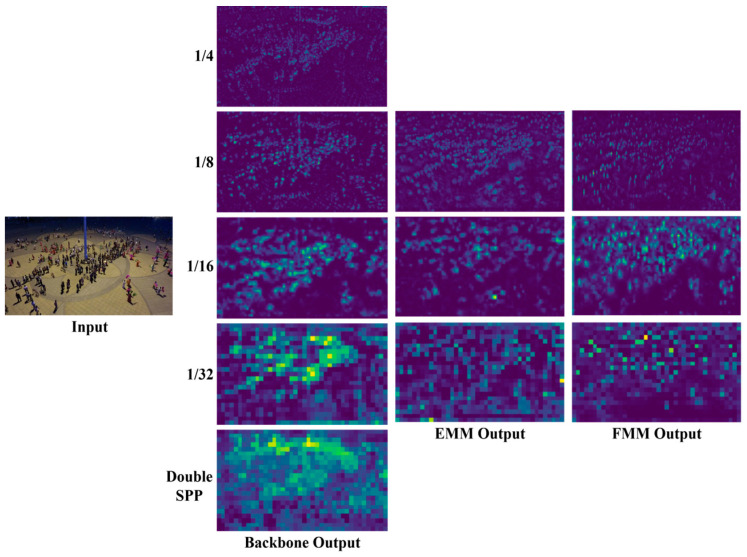
The visualization of HRMamba-YOLO.

In addition, as shown in Figure 9, we also provide the visualization results of the feature maps output by the backbone (including our proposed Double SPP module), as well as the visualization results of the feature maps output by EMM and FMM. From the figure, it can be seen that with the combined effects of the Double SPP, EMM, and FMM modules, the detection of objects becomes increasingly precise.

## 4. Results

### 4.1. Datasets

**Figure 10:**
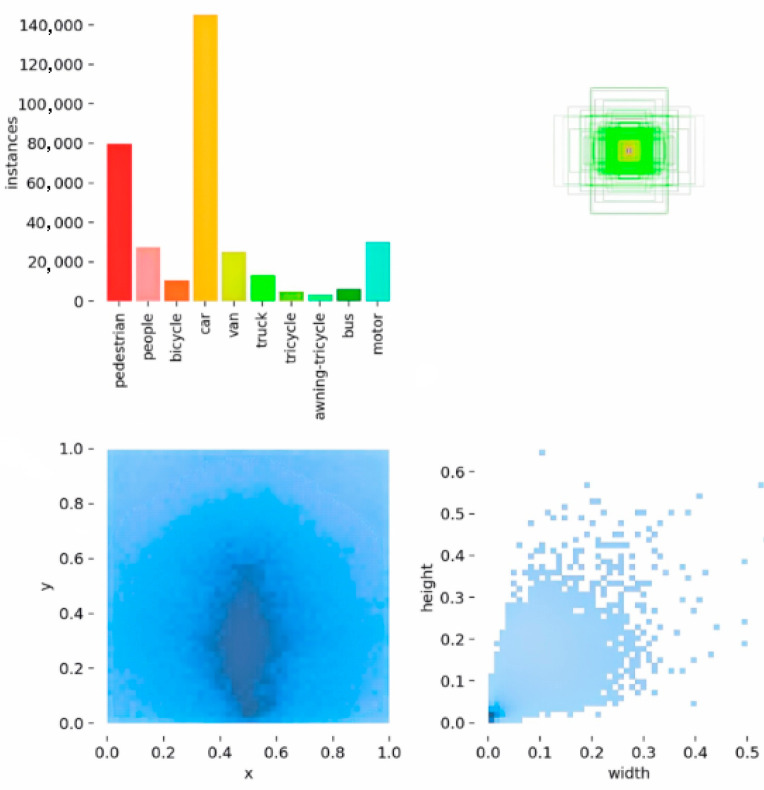
The Visdrone2019 datasets.

The VisDroneDET 2019 dataset presents significant challenges in the field of object detection due to its characteristics such as scale variation, occlusion issues, and class imbalance. The dataset consists of 8629 images, distributed according to the following: 6471 images for the training set, 548 for the validation set, and 1610 for the test set [4]. These images were captured by various cameras mounted on drones across 14 different cities in China, spanning thousands of kilometers and covering diverse environments ranging from urban to rural areas. The dataset encompasses a wide variety of object types, including pedestrians, vehicles, bicycles, and more, with varying scene densities from sparse to crowded. Additionally, the collection of this dataset involved multiple drone platforms and was conducted under different scenarios, weather conditions, and lighting to achieve diverse data acquisition. Figure 10 shows the annotation, location, and size distribution of images in the Visdrone2019 datasets.

### 4.2. Implementation Details

In terms of implementation details, this research builds upon the YOLOv8 model with a series of improvements. Specifically, the input image resolution was set to 1280 × 1280, and the Mosaic data augmentation technique was employed. The training process lasted for 120 epochs, with an initial learning rate of 0.01, using SGD as the optimizer and setting the momentum parameter to 0.937. Throughout the training process, we used the Geforce Gigabyte RTX 3090 GPU (Shenzhen Whale Spring Technology Co., Ltd., Shenzhen, China) and ensured consistency in environmental settings and random seeds to guarantee the reproducibility of the experiments and the accuracy of the results.

### 4.3. Evaluation Metrics

Precision: This metric evaluates the proportion of actual positives among the samples predicted as positive by the model. The formula for precision is

(9) $$Precision=\frac{TP}{TP+FP}$$

In this formula, True Positives (TPs) represent the number of samples correctly identified as positive, while False Positives (FPs) refer to those samples that are incorrectly classified as positive when they are actually negative. A high level of precision reflects the model’s accuracy and reliability in discriminating positive samples.

Recall measures the proportion of actual positive samples that the model successfully identifies, indicating the model’s ability to recognize true positives. The recall calculation formula is

(10) Recall=TPTP+FN

False Negatives (FNs) indicate the number of actual positive samples that the model incorrectly labels as negative.

AP stands for Average Precision (AP), which is simply the mean of the Precision values on the Precision–Recall (PR) curve. We used integration to calculate the PR curve. The AP formula is

(11) $$\mathrm{AP}=\int_{0}^{1}p(r)d_{r}$$

In practical applications, the PR curve is not directly calculated. Instead, we smoothed the PR curve by taking the maximum precision value to the right of each point on the curve.

The mean Average Precision (mAP) measures the overall performance of a model in multi-class detection tasks and is the arithmetic mean of the AP values for all categories. The calculation of the mAP is described as

(12) $$\mathrm{mAP}=\frac{1}{N}\sum_{i=1}^{N}AP_{i}$$

Here, AP_i_ represents the average precision score for the i-th category, while N is the total number of categories. A high mAP value indicates that a model performs with high precision and recall on average across all categories, making it a key metric for evaluating a model’s comprehensive performance in multi-class recognition tasks.

### 4.4. Comparison of State-of-the-Art Methods

Table 1 presents a quantitative comparison on the Visdrone dataset with existing state-of-the-art methods. The results show that HRMamba-YOLO outperforms other methods in mAP metrics. Notably, this new algorithm in the YOLO series maintains good performance in detecting small objects while keeping the model lightweight. HRMamba-YOLO achieves the best detection results while maintaining real-time inference, demonstrating its effectiveness and efficiency.

### 4.5. Ablation Experiments and Analysis

(1) Ablation experiments on Double SPP

In this section, we conducted a series of ablation experiments to evaluate the effectiveness of the Double SPP module on the Visdrone dataset. The experiments used the original YOLOv8-m as the baseline model and gradually incorporated different enhancements. Specifically, we compared the following four variants:

- Variant A—Utilizing the original single-branch SPP structure;
- Variant B—Adding an SE module after the single-branch SPP;
- Variant C—Introducing a second SPP branch;
- Variant D—A dual-branch SPP structure with an SE module added to one branch.

As shown in Table 2, both Variants B and C showed improvements over A, indicating that the SE module and the additional SPP branch contributed to performance enhancement. More importantly, Variant D outperformed all other variants across all performance metrics, demonstrating that the Double SPP structure effectively enhanced feature extraction capabilities. Although there was an increase in computational load and latency (from Variants A to D, latency from 12.3 ms to 12.9 ms), these increases were within acceptable limits. These results confirm that the introduction of Double SPP significantly contributes to model performance improvement.

(2) Ablation experiments on EMM

To validate the efficacy of the EMM module, we performed ablation experiments using the modified YOLOv8-m with the Double SPP as the baseline. The EMM module is designed to enhance features prior to the feature fusion network by capturing and integrating high-resolution feature information into lower-resolution features. This module models both global (using the Mamba technique) and local (using the DWConv technique) perspectives for broader contextual information capture. As indicated in Table 3, the experimental results show the following:

- When only global processing (Mamba technique) was used, there was a performance increase, despite a decrease in inference speed.
- When only local processing (DWConv technique) was used, performance also improved, albeit to a lesser extent than global processing.
- When both global and local processing were employed, there was a significant uplift in overall model performance, albeit at the cost of further reduced inference speed.

These results confirm the effectiveness of the EMM module, particularly when global and local processing are combined, significantly enhancing the detection performance of the model. While there is a trade-off in inference speed, the performance gains suggest that this compromise is worthwhile, especially in applications that demand high detection accuracy.

(3) Effectiveness of Multi-Scale Input in FMM

To validate the effectiveness of multi-scale input in the FMM module, we conducted a series of comparative experiments. The FMM module’s input includes not only the features of the current scale but also auxiliary feature information from two other distinct scales. This design can be seen as an enhancement to the HRFPN, achieving additional feature fusion and strengthening the feature representation capability. Through the ES2D mechanism, FMM can reconstruct global features containing extensive contextual information, while the nonlinear transformation applied by the FFN further enhances the model’s representational power.

We designed two experimental setups:

- With auxiliary input—Utilizing multi-scale feature information in FMM;
- Without auxiliary input—Using only the feature information of the current scale.

The experimental results, as shown in Table 4, indicate that the input of multi-scale feature information in FMM is effective.

(4) Structural Analysis of HRFPN

In this section, we delve into the analysis of HRFPN and three other feature fusion network structures for comparison. Table 5 demonstrates that, for small object detection tasks, the HRNet style feature fusion network significantly surpasses the performance of the PANet used in YOLOv8. Furthermore, the integration of additional upsampling and concatenation operations into the HRNetv2 style network, while slightly increasing inference latency, notably enhances network performance.

(5) Step-by-Step Ablation Experiments

In this experiment, the YOLOv8-m model was selected as the baseline (Variant A) for study. Initially, the original SPP module was replaced with a Double SPP module, significantly enhancing model performance. Building upon Variant B, the EMM (Variant C) and FMM (Variant D) were integrated, with the results indicating that the Mamba structure and the fusion of multi-scale feature information markedly improved small object detection performance. Furthermore, substituting YOLOv8′s feature fusion network PANet with HRFPN (Variant F) resulted in a notable increase in model accuracy. Combination experiments of EMM, FMM, and HRFPN (Variants E, G, and H) demonstrated the efficacy of these integrated modules. Ultimately, the final model, named HRMamba-YOLO (Variant I), incurred a slight increase in inference time, from 12.3 milliseconds for Variant A to 31.1 milliseconds; however, the mean Average Precision (mAP) rose dramatically from 30.8% to 35.5% (Table 6).

### 4.6. Visualization and Detection Results

**Figure 11:**
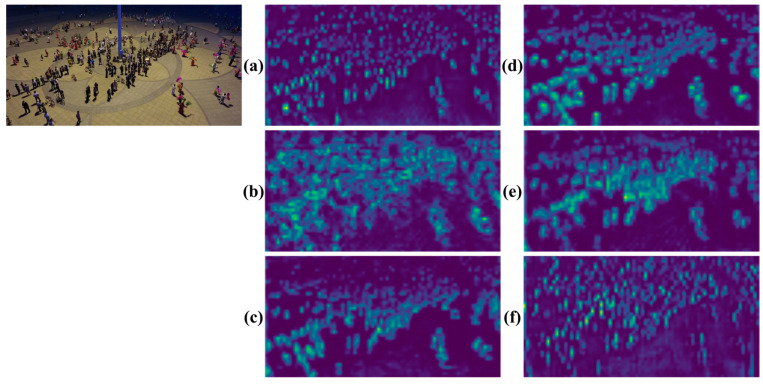
Visualization of the feature map with one-eighth resolution (here, (**a**–**f**) represent YOLOv5-m, YOLOv6-m, YOLOv7, YOLOv8-m, YOLOX-m, and HRMamba-YOLO, respectively).

**Figure 12:**
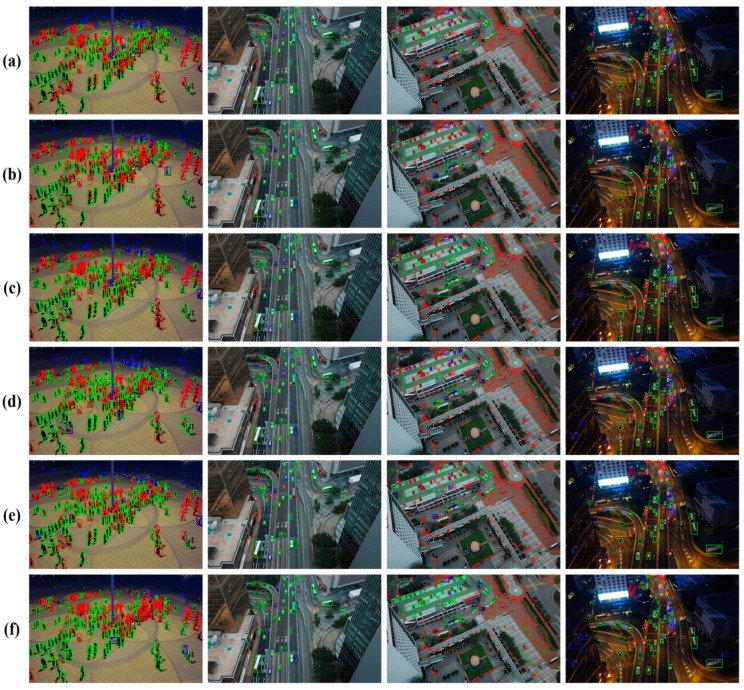
Comparison of the detection results (Visdrone2019; here, (**a**–**f**) represent YOLOv5-m, YOLOv6-m, YOLOv7, YOLOv8, YOLOX-m, and HRMamba-YOLO, respectively, and the same applies to the following figures).

Figure 11 shows the feature map visualization results of several algorithms. It can be seen from the figure that the visualization results of the proposed algorithm HRMamba-YOLO are clearer. To clearly demonstrate the detection results, differently colored boxes were used to distinguish various detection scenarios: green boxes indicate TP, where both classification and localization were accurate; blue boxes symbolize FP, indicating errors in classification or localization; and red boxes represent FN, where both classification and localization errors occurred, or the target was not detected. Figure 12 vividly displays these results, clearly revealing HRMamba-YOLO’s superior classification and localization abilities on small object datasets. HRMamba-YOLO was not only capable of capturing numerous small objects that YOLOv8-m failed to recognize but also demonstrated remarkable performance in detecting densely packed crowds in the first image, showcasing its high detection precision and efficiency.

Table 7 presents a comparison of the mAP values for different classes in YOLOv5-m, YOLOv6-m, YOLOv7, YOLOv8-m, YOLOX-m, and the proposed method, HRMamba-YOLO. It can be seen from the figure that the proposed method achieved the highest mAP detection results.

### 4.7. Expansion Experiment

The Dota1.5 dataset is a remote-sensing image dataset used for object detection and tracking. The dataset is mainly used for object detection and tracking research in UAV scenarios. The Dota1.5 dataset is an extension and improvement of the original Dota dataset to better meet the needs of UAV visual tasks. This dataset includes UAV images in different environmental conditions, covering various real-world object categories, providing rich data resources for related research.

In Table 8 and Table 9, we present a comparison of the detection results for various methods in the Dota1.5 dataset. It can be seen from the figure that the proposed algorithm achieved the highest mAP detection value.

**Figure 13:**
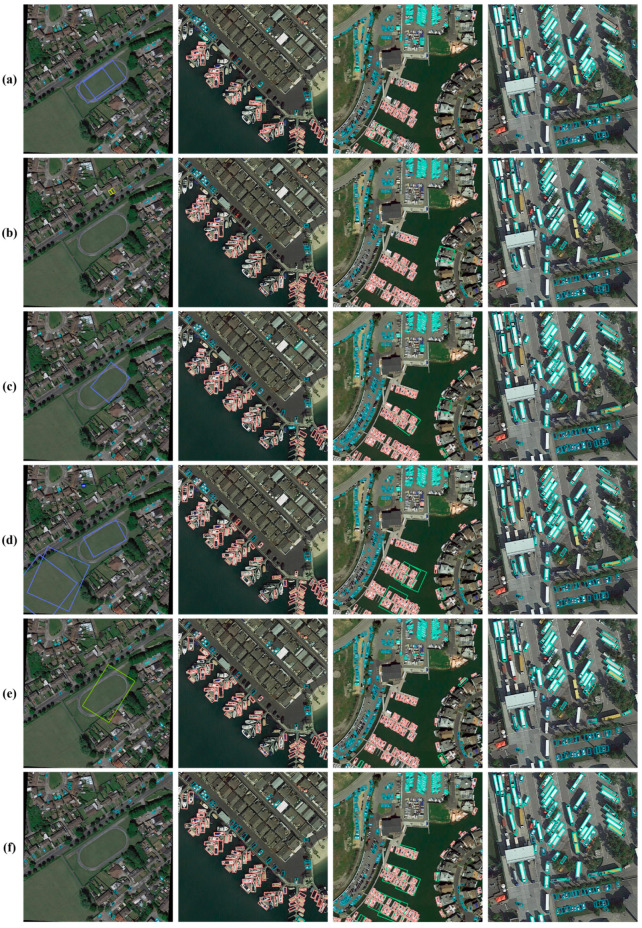
Comparison of the detection results (Dota1.5, here, (**a**–**f**) represent YOLOv5-m, YOLOv6-m, YOLOv7, YOLOv8, YOLOX-m, and HRMamba-YOLO, respectively).

From the comparison of the detection results in Figure 13 for algorithms YOLOv5-m(a), YOLOv6-m(b), YOLOv7(c), YOLOv8-m(d), and YOLOX-m(e), it is evident that the proposed HRMamba-YOLO(f) algorithm provided the most accurate detection and could identify more small objects. The UCAS-AOD dataset is a remote-sensing image dataset mainly used for object detection and recognition tasks. The dataset includes two types of objects, cars and airplanes, as well as background negative samples. Table 10 gives the mAP comparison for these two classes. The dataset for vehicles comprises 310 images containing 2819 vehicle samples, while the dataset for planes consists of 600 images with 3210 plane samples. The samples were meticulously chosen to ensure even distribution of object orientations in the datasets. Each dataset was divided into two subsets: (250 images, 60 images) and (500 images, 100 images). One subset was allocated for training, while the other was designated for testing.

The research team led by Han Junwei from Xi’an University of Technology proposed the large-scale benchmark dataset “DIOR” for object detection in optical remote-sensing images. The dataset consists of 23,463 images and 190,288 object instances. Table 11, Table 12 and Table 13 give mAP comparisons for different classes in the DIOR dataset.

## 5. Conclusions

We introduced a small object detection algorithm named HRMamba-YOLO, which effectively detects small objects by combining the strengths of HRNet, EfficientVMamba, and YOLO. This study opened by incorporating a dual-branch SPP structure with an SE attention module integrated into one branch, significantly enhancing the network’s capacity to extract features from small objects, thus improving detection precision. Then, based on HRNet, an HRFPN was designed to enhance the efficiency of small object detection through cross-scale and within-scale feature fusion, while maintaining high-resolution feature maps conducive to small object recognition. Subsequently, EMM and FMM were developed utilizing multi-scale feature fusion techniques and an ES2D selective scanning mechanism, reinforcing the interaction between features and boosting the model’s performance in small object detection. Experiments on the VisDroneDET dataset demonstrated that HRMamba-YOLO outperformed existing methods in mAP and latency metrics, proving its effectiveness and efficiency in small object detection tasks.

For the VisDroneDET dataset, the proposed algorithm achieved a 4.4% higher mAP compared to YOLOv8-m. The experimental results showed that HRMamba achieved a mAP of 37.1%, surpassing YOLOv8-m by 3.8% (Dota1.5 dataset). For the UCAS_AOD dataset and the DIOR dataset, our model had a mAP 1.5 percent and 0.3 percent higher than the YOLOv8-m model, respectively. To be fair, all the models were trained without a pre-trained model. This consistent improvement may indicate that HRMamba-YOLO has better performance and universality in handling drone images. These results could be of significant inspiration for research and practice in the field of drone vision.

## Figures and Tables

**Table 1:** Comparison of state-of-the-art methods.

Method	mAP (%)	Latency (ms)
YOLOv3-tiny [26]	15.9	3.2
YOLOv4-tiny [1]	27.6	20
YOLOv5-s [5]	29.1	10.8
YOLOv5-m [5]	33.9	22.1
YOLOv6-s [6]	30.2	12.8
YOLOv6-m [6]	33.7	21.9
YOLOv7 [2]	36.3	34.6
YOLOv7-tiny [2]	29.8	7.5
YOLOv8-s [5]	34.8	12.3
YOLOv8-m [5]	34.5	26.9
YOLOX-s [7]	32.7	11.5
YOLOX-m [7]	34.2	24.6
Faster-RCNN [38]	21.4	–
CenterNet [39]	29.1	–
DMNet [40]	28.7	–
SSD [3]	25.3	–
ClusDet [41]	31.7	–
DREN [42]	30.3	–
GLSAN [24]	32.5	–
QueryDet [43]	28.3	–
**HRMamba-YOLO (Ours)**	**38.9**	**31.1**

**Table 2:** Ablation experiments on Double SPP.

Variant	SPP’s Number	SE	mAP (%)	Latency (ms)
A	1		34.8	12.3
B	1	✓	34.9	12.5
C	2		34.9	12.7
D	2	✓	35.1	12.9

**Table 3:** Ablation experiments on EMM.

Global	Local	Param (M)	GFLOPs	mAP (%)	Latency (ms)
		15.2	32.1	35.1	12.9
✓		17.6	35.1	35.6	17.4
✓	✓	16.9	35.2	35.3	16.4
	✓	17.7	35.4	35.9	18.7

**Table 4:** Experiment to validate the effectiveness of multi-scale input (FMM).

Method	mAP (%)	Latency (ms)
w/Auxiliary Input	36.4	25.5
w/o Auxiliary Input	35.6	24.7

**Table 5:** Comparison experiment of different feature fusion networks.

Method	Param (M)	GFLOPs	mAP (%)	Latency (ms)
PANet	15.2	32.1	35.1	12.9
HRNetv1 style	19.4	42.5	35.9	19.2
HRNetv2 style	14.9	35.5	36.2	16.2
HRFPN	15.1	36.8	36.6	16.4

**Table 6:** The step-by-step ablation experiment for HRMamba-YOLO.

Variant	Double SPP	PANet	HRFPN	EMM	FMM	Param (M)	GFLOPs	mAP (%)	Latency (ms)
A		✓				11.2	28.8	34.8	12.3
B	✓	✓				15.2	32.1	35.1	12.9
C	✓	✓		✓		17.7	35.4	35.9	18.7
D	✓	✓			✓	31.6	90.3	36.4	25.5
E	✓	✓		✓	✓	34.1	93.6	37.4	27.4
F	✓		✓			15.1	36.8	36.6	16.4
G	✓		✓	✓		17.6	39.9	38.2	23
H	✓		✓		✓	31.4	94.8	38.5	29.8
I	✓		✓	✓	✓	33.5	96.4	38.9	31.1

**Table 7:** A mAP comparison of different classes in Visdrone2019.

Model	mAP (%)	Pedestrian	People	Bicycle	Car	Van	Truck	Tricycle	Awning-Tricycle	Bus	Motor
YOLOv5-m [5]	33.9	34.1	23.8	17.3	64.6	41.2	35.0	25.4	16.7	49.0	31.7
YOLOv6-m [6]	33.7	32.8	23.1	17.0	64.0	41.4	35.5	26.1	17.0	49.8	30.6
YOLOv7 [2]	36.3	36.9	25.4	19.7	66.8	44.4	37.6	26.2	16.7	55.5	33.7
YOLOv8-m [5]	34.5	34.6	23.9	17.8	65.1	42.0	36.5	25.4	16.1	51.4	32.2
YOLOX-m [7]	34.2	34.7	24.3	17.9	65.5	42.3	33.5	25.0	17.1	49.3	32.0
HRMamba-YOLO (Ours)	38.9	37.5	26.8	21.6	68.2	46.8	41.6	30.9	19.9	58.9	36.8

**Table 8:** The mAP comparison for different classes in Dota1.5 (part 1).

Method	mAP (%)	Plane	Ship	Storage Tank	Baseball Diamond	Tennis Court	Basketball Court	Ground Track Field
YOLOv5-m [5]	31.1	58.9	44.1	38.5	27.2	74.0	20.2	19.2
YOLOv6-m [6]	32.0	60.2	44.9	39.5	29.6	77.9	24.2	21.1
YOLOv7 [2]	34.4	63.5	48.2	43.6	32.1	78.1	23.8	19.5
YOLOv8-m [5]	33.3	60.3	45.8	40.6	31.1	78.1	22.7	22.9
YOLOX-m [7]	30.7	60.2	45.2	39.6	29.9	72.0	18.8	15.4
HRMamba-YOLO (Ours)	37.1	64.6	48.2	39.6	39.3	83.0	25.8	26.0

**Table 9:** The mAP comparison for different classes in Dota1.5 (part 2).

Method	mAP (%)	Bridge	Large Vehicle	Small Vehicle	Helicopter	Roundabout	Soccer Ball Field	Swimming Pool
YOLOv5-m [5]	31.1	2.7	56.0	34.6	14.1	9.2	18.5	19.6
YOLOv6-m [6]	32.0	2.5	55.7	36.0	16.7	8.5	18.7	15.5
YOLOv7 [2]	34.4	5.0	56.3	36.0	24.3	15.1	20.9	20.7
YOLOv8-m [5]	33.3	3.7	55.7	35.3	19.9	11.4	21.7	19.8
YOLOX-m [7]	30.7	2.2	55.7	34.6	14.2	10.5	18.1	17.3
HRMamba-YOLO (Ours)	37.1	6.9	59.7	38.8	26.5	15.1	19.0	28.0

**Table 10:** The mAP comparison for different classes in UCAS-AOD.

Method	mAP (%)	Plane	Car
YOLOv5-m [5]	64.5	72.7	56.4
YOLOv6-m [6]	64.7	72.4	57.1
YOLOv7 [2]	64.6	70.9	58.3
YOLOv8-m [5]	65.2	71.9	58.5
YOLOX-m [7]	63.9	72.2	55.5
HRMamba-YOLO (Ours)	66.7	73.2	60.1

**Table 11:** The mAP comparison for different classes in DIOR (part 1).

Method	mAP (%)	Airplane	Airport	Baseball Field	Basketball Court	Bridge	Chimney	Dam
YOLOv5-m [5]	56.3	72.1	50.3	78.4	77.2	27.5	72.6	36.0
YOLOv6-m [6]	55.0	71.9	47.7	77.5	76.1	25.4	73.6	34.2
YOLOv7 [2]	58.8	73.9	60.0	79.8	78.9	29.2	75.5	37.4
YOLOv8-m [5]	65.8	78.2	70.7	82.4	83.3	37.4	81.7	53.1
YOLOX-m [7]	64.6	77.4	66.4	82.1	82.1	38.3	80.0	50.1
HRMamba-YOLO (Ours)	66.1	79.1	71.4	83.0	83.4	38.1	81.0	51.7

**Table 12:** The mAP comparison for different classes in DIOR (part 2).

Method	mAP (%)	Expressway Service Area	Expressway toll Station	Golf Field	Ground Track Field	Harbor	Overpass	Ship
YOLOv5-m [5]	56.3	62.9	51.2	59.7	70.1	45.8	42.2	57.0
YOLOv6-m [6]	55.0	60.4	52.3	53.3	69.3	42.5	40.7	55.7
YOLOv7 [2]	58.8	65.1	55.7	64.9	72.5	46.5	42.6	57.6
YOLOv8-m [5]	65.8	75.1	65.8	75.2	78.3	55.3	50.5	61.5
YOLOX-m [7]	64.6	74.6	64.4	71.1	76.9	55.4	48.9	61.0
HRMamba-YOLO (Ours)	66.1	75.7	66.0	75.4	78.1	55.3	50.5	61.5

**Table 13:** The mAP comparison for different classes in DIOR (part 3).

Method	mAP (%)	Stadium	Storage Tank	Tennis Court	Train Station	Vehicle	Windmill
YOLOv5-m [5]	56.3	76.7	56.7	83.7	25.4	34.1	45.8
YOLOv6-m [6]	55.0	74.7	53.6	82.8	27.5	33.3	46.6
YOLOv7 [2]	58.8	77.8	57.7	84.6	33.2	36.0	47.7
YOLOv8-m [5]	65.8	84.6	60.9	87.0	41.0	40.5	54.3
YOLOX-m [7]	64.6	83.1	60.5	86.4	41.2	40.1	52.9
HRMamba-YOLO (Ours)	66.1	86.4	61.3	87.1	41.4	40.9	55.2

## Data Availability

The original data presented in this study are openly available at https://github.com/VisDrone/VisDrone-Dataset, (accessed on 24 September 2023); https://captain-whu.github.io/DOAI2019/dataset.html, (accessed on 20 May 2024); and https://hyper.ai/datasets/5419, https://pan.baidu.com/s/1w8iq2WvgXORb3ZEGtmRGOw, (accessed on 20 May 2024).
